# The Production of Monokaryotic Hyphae by *Cryptococcus neoformans* Can Be Induced by High Temperature Arrest of the Cell Cycle and Is Independent of Same-Sex Mating

**DOI:** 10.1371/journal.ppat.1003335

**Published:** 2013-05-02

**Authors:** Jianmin Fu, Ian R. Morris, Brian L. Wickes

**Affiliations:** The Department of Microbiology and Immunology, The University of Texas Health Science Center at San Antonio, San Antonio, Texas, United States of America; Washington University School of Medicine, United States of America

## Abstract

*Cryptococcus neoformans* is a heterothallic fungal pathogen of humans and animals. Although the fungus grows primarily as a yeast, hyphae are produced during the sexual phase and during a process called monokaryotic fruiting, which is also believed to involve sexual reproduction, but between cells of the same mating type. Here we report a novel monokaryotic fruiting mechanism that is dependent on the cell cycle and occurs in haploid cells in the absence of sexual reproduction. Cells grown at 37°C were found to rapidly produce hyphae (∼4 hrs) and at high frequency (∼40% of the population) after inoculation onto hyphae-inducing agar. Microscopic examination of the 37°C seed culture revealed a mixture of normal-sized and enlarged cells. Micromanipulation of single cells demonstrated that only enlarged cells were able to produce hyphae and genetic analysis confirmed that hyphae did not arise from *α-α* mating or endoduplication. Cell cycle analysis revealed that cells grown at 37°C had an increased population of cells in G2 arrest, with the proportion correlated with the frequency of monokaryotic fruiting. Cell sorting experiments demonstrated that enlarged cells were only found in the G2-arrested population and only this population contained cells able to produce hyphae. Treatment of cells at low temperature with the G2 cell cycle arrest agent, nocodazole, induced hyphal growth, confirming the role of the cell cycle in this process. Taken together, these results reveal a mating-independent mechanism for monokaryotic fruiting, which is dependent on the cell cycle for induction of hyphal competency.

## Author Summary

Fungi typically grow vegetatively as either yeast or hyphae. Many of the major human fungal pathogens can generate both morphologies and are referred to as the dimorphic fungi. *Cryptococcus neoformans* is a yeast-like fungus that has not been traditionally thought to be dimorphic since hyphae production typically occurs during the mating process between cells of opposite mating types. However, *C. neoformans* also can generate the hyphal state from haploid cells (called monokaryotic or haploid fruiting) in the absence of the opposite mating type. Recent studies have shown that the mechanism behind this process also involves mating, however, the mating reaction occurs between cells of the same mating type. Here we describe a unique mechanism responsible for monokaryotic fruiting that is independent of mating and does not proceed through a diploid intermediate. Instead, the key requirement for hyphal induction appears to be cell cycle arrest. Importantly, arrested cells display an enlarged cell phenotype, which has been observed *in vivo* in recent reports and has been hypothesized to be a novel protection strategy against host defenses. *C. neoformans* appears to have an extensive morphological repertoire, which likely contributes to its success as both a pathogen and a saprophyte.

## Introduction

*Cryptococcus neoformans* is a basidiomycetous fungal pathogen of humans and animals that typically causes opportunistic infections in patients with cellular immune defects [1]. Infection initiates in the lungs and frequently disseminates to the brain where it manifests as a fatal meningoencephalitis if untreated. AIDS patients are at increased risk for infection, though infection rates have decreased significantly with better AIDS management [2]. However, in spite of the reduction in AIDS-related cases, cryptococcosis remains a frequent life-threatening opportunistic mycosis for these patients in underdeveloped countries, and is a recently emergent disease in the United States Pacific Northwest [3] for as yet, unexplained reasons.

Naturally occurring strains of *C. neoformans* are heterothallic with two mating types, *MATa* and *MATα*, with both mating types being pathogenic, although most clinical isolates are *MATα* [4]. Although the taxonomy has been changing, four serotypes have been described (A, B, C, D) with serotypes A and D often referred to as *C. neoformans* variety *grubii* and variety *neoformans* respectively, and serotypes B and C being collectively referred to as *C. neoformans* variety *gattii*, or more recently, *C. gattii* [5]. For all serotypes, throughout the course of infection and under normal culture conditions, the fungus grows as an encapsulated yeast. Under appropriate *in vitro* conditions, however, the fungus can produce two kinds of hyphae; dikaryotic hyphae during *MATa*×*MATα* sexual reproduction and monokaryotic hyphae (from individual *MATa* or *MATα* strains) during monokaryotic fruiting [6]. Basidiospores can be produced from both hyphal types. Environmental factors required for sexual reproduction and monokaryotic fruiting are similar and include culture under low temperature (25°C), low moisture, and nutrient limitation [6]. Many genes, including homologs of the *Saccharomyces cerevisiae* pheromone response pathway, are required to produce both types of hyphae [7], [8], [9], [10], [11]. There are, however, distinct differences between the two hyphal types. Structurally, dikaryotic hyphae have fused clamp connections and a pair of nuclei (one *MATa* and one *MATα*) per hyphal compartment while monokaryotic hyphae have unfused clamp connections and a single nucleus per hyphal compartment. Sexual reproduction in *C. neoformans* has been characterized in detail and largely follows the pheromone response paradigm that has been developed from decades of *S. cerevisiae* research [12]. Less clear is the mechanism by which monokaryotic fruiting occurs.

Recent studies have concluded that monokaryotic fruiting in *C. neoformans* variety *neoformans* can result from *α*-*α* mating [13] and may be an important part of the natural life cycle of this fungus [14], with possible implications for human disease [15]. We recently reported that high temperature seed culture conditions could induce very robust monokaryotic fruiting in *C. neoformans* variety *neoformans* [11] and assumed these growth conditions enhanced *α*-*α* cell fusion. However, this assumption proved to be false. Instead, we found that cells arrested in the G2 stage of the cell cycle were competent to undergo monokaryotic fruiting at high frequency, in the absence of *α*-*α* cell fusion. Importantly, this mechanism proceeds through enlarged cells, a morphological phenotype that has been increasingly observed *in vivo* and is hypothesized to serve as a strategy for avoiding host defenses [16], [17], [18]. These results demonstrate that *C. neoformans* has evolved a number of different mechanisms for modifying cellular morphology to suit its specific environment, with some of these mechanisms contributing to the success of this fungus as a pathogen.

## Results

### Seed culture conditions determine competence for monokaryotic fruiting

**Figure 1:**
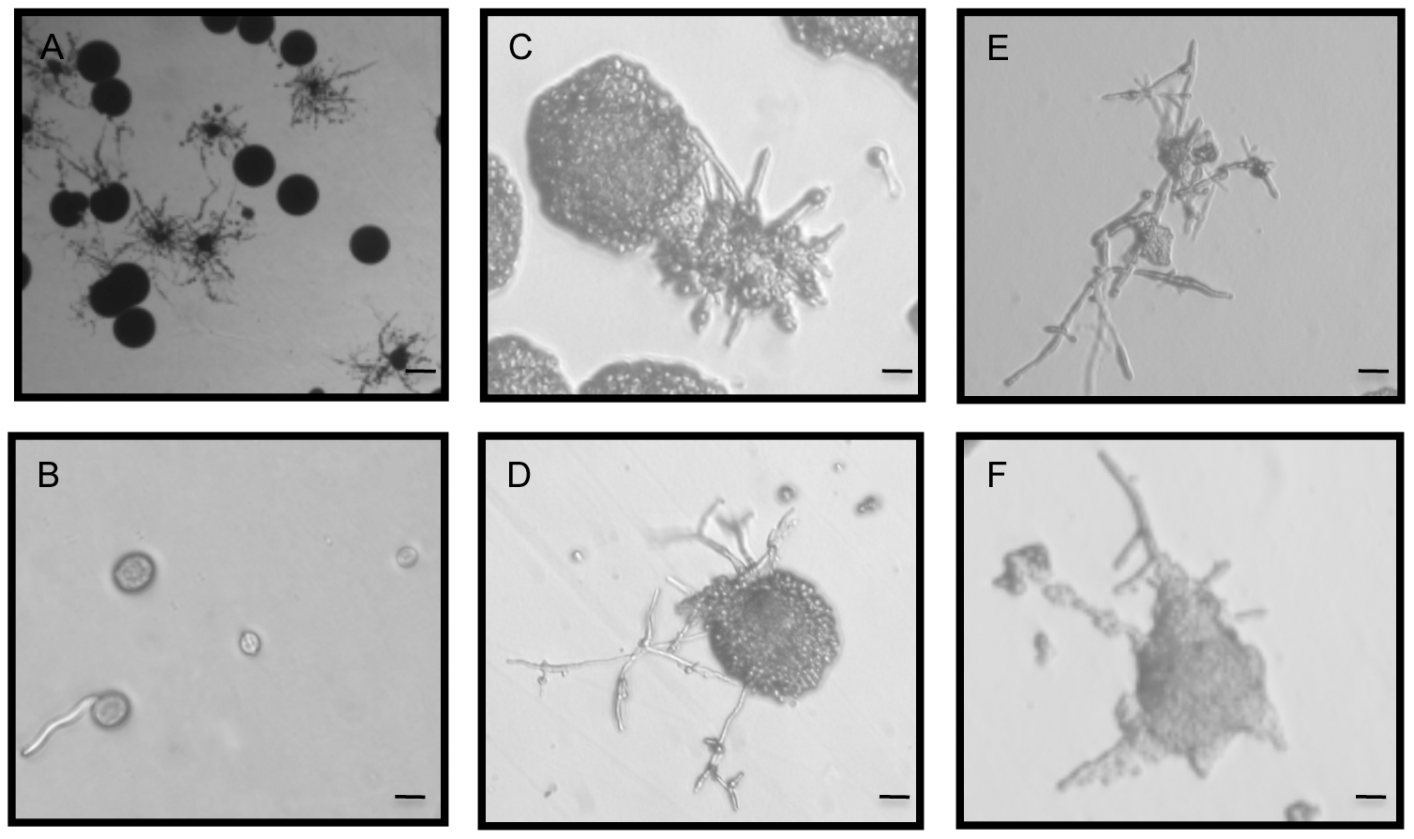
Monokaryotic fruiting on filament agar. (A) Individual colonies visualized at 2× on a spread plate after 4 days growth at 25°C from a seed culture (JEC-21) inoculum grown at 37°C for 24 h on YPD. Bar = 1 mm. (B) Individual cells under the same seed culture and plating conditions as in (A), but visualized after 4 h at 200× magnification. Bar = 10 µm. (C–F) Monokaryotic fruiting in all serotypes of *C. neoformans*. (C) WSA-79 (serotype D, also known as *C. neoformans* variety *neoformans*), (D) WSA-522 (serotype A, also known as *C. neoformans* variety *grubii*), (E) WSA-533 (serotype B, also known as *C. neoformans* variety *gattii*, or *C. gattii*), (F) WSA-2507 (serotype C, also known as *C. neoformans* variety *gattii*, or *C. gattii*). Colonies visualized at 200×. Bar = 10 µm.

Previous studies of monokaryotic fruiting typically were performed by patching cells from a seed culture onto filament agar and then screening for a hyphal fringe around the periphery [6], [11], [19]. Our recent observation of the role of temperature in this process [11] led us to test whether or not high temperature increases the intensity of hyphae production after inoculation onto filament agar, or increases the number of cells that produce hyphae. A suspension of cells from a 24 h, 37°C seed culture was spread onto filament agar to observe monokaryotic fruiting in individual cells, which would reveal whether or not all cells from the seed culture were capable of undergoing monokaryotic fruiting. Figure 1A demonstrates that only part of the population grown at 37°C was able to undergo monokaryotic fruiting and that hyphae began to appear as early as 4 hours after plating onto filament agar (Fig. 1B). This phenomenon was not a strain artifact nor was it restricted to a single serotype as all four serotypes were found to be able to produce hyphae under the above inducing conditions (Fig. 1C–F). These results suggest that only a specific type of cell is capable of undergoing monokaryotic fruiting, and that this capability requires seed culture conditions that enable these cells to become competent for hyphal production.

### Temperature-induced hyphal competency does not result from *α*-*α* cell fusion

Lin *et al.*, have demonstrated that same-sex mating is one way in which monokaryotic fruiting can occur [13]. However, because the previous experiment showed that individual cells were still capable of monokaryotic fruiting in spite of being well separated from potential mating partners on spread plates, we hypothesized that either same-sex mating occurs during the seed culture growth period, or that another mechanism, which does not involve same-sex mating, could also lead to monokaryotic fruiting.

**Figure 2:**
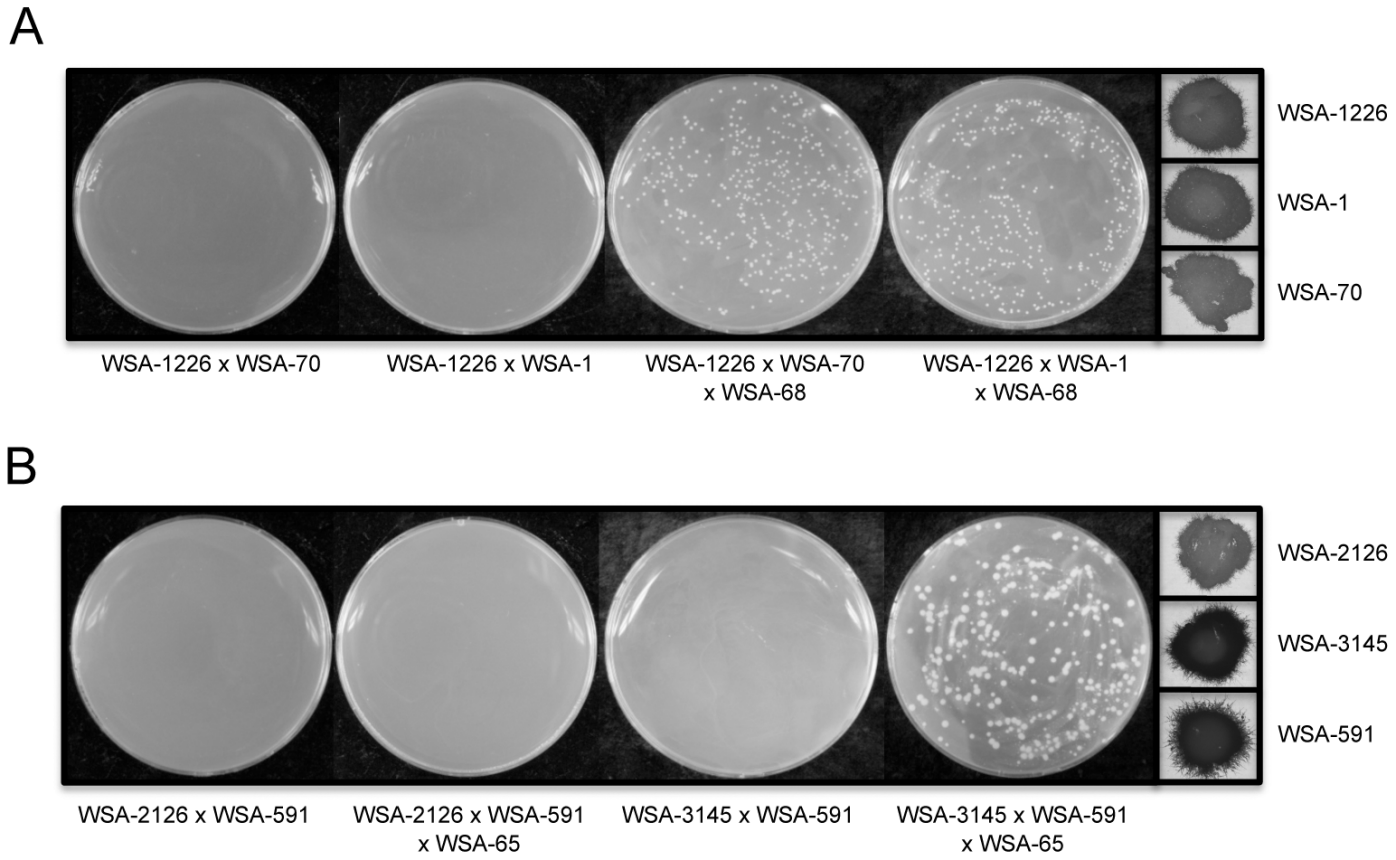
Exclusion of *α*-*α* cell fusion as a mechanism for monokaryotic fruiting. (A) Crosses between *MATα* strains with complementary auxotrophic markers do not yield fusants (WSA-1226×WSA-70, or WSA-1226×WSA-1) unless the mating is assisted with a *MATa* (WSA-68) helper strain (WSA-1226×WSA-70×WSA-68, or WSA-1226×WSA-1×WSA-68). However, the three *MATα* strains are capable of monokaryotic fruiting (insets). (B) To rule out an undetectable fusion event as an explanation for the results seen in (A), a fusion defective strain (WSA-2126) was included in the assay. This strain, which is a *cpk1* mutant, did not yield fusants with WSA-591 alone or with a *MATa* (WSA-65) helper. Complementation of WSA-2126 with the *CPK1* gene (WSA-3145) did not yield fusants with WSA-591 unless WSA-65 was included as a helper. However, all three *MATα* strains were capable of monokaryotic fruiting, including the *cpk1* mutant (inserts) ruling out *α*-*α* cell fusion as the mechanism for monokaryotic fruiting.

To investigate these two possibilities, we first screened for evidence of *α*-*α* mating during monokaryotic fruiting using the *α*-*α* cell fusion assay to test different combinations of complementing *MATα* auxotrophs that would be predicted to yield prototrophic colonies on unsupplemented MIN agar. No fusants were observed from *MATα*×*MATα* crosses plated onto MIN agar after growth as a seed culture on YPD at 37°C for 24 h. However, assisted *α*-*α* matings showed that each of these strains was capable of undergoing *α*-*α* fusion, ruling out an *α*-*α* cell fusion defect (Fig. 2A). The most likely explanation for these results was that because *α*-*α* fusion is a rare event [13], our conditions, although not detecting an *α*-*α* fusion, were still not excluding this possible mechanism. To exclude a cryptic fusion event as an explanation for our observations, we utilized a *cpk1Δ* mutant to further test whether or not *α*-*α* fusion was required for temperature-induced monokaryotic fruiting. Cpk1p, the MAP kinase in the *C. neoformans* pheromone response pathway, is required for *α-a* fusion during sexual reproduction as well as *α*-*α* fusion during monokaryotic fruiting [9], [13]. Therefore, if *α*-*α* fusion was the mechanism of monokaryotic fruiting in our system, the *cpk1Δ* mutant should not produce hyphae after plating onto filament agar because it could not fuse with the complementing strain. Our results showed that neither assisted nor unassisted *α*-*α* mating reactions (WSA-2126×WSA-591×WSA-65 or WSA-2126×WSA-591) with the *cpk1Δ* mutant (WSA-2126) showed evidence of *α*-*α* cell fusion (Fig. 2B). However, when WSA-2126 was tested for monokaryotic fruiting ability after high temperature seed culture, this strain fruited normally (Fig. 2B). These results confirmed that monokaryotic fruiting could occur independently of *α*-*α* fusion.

### Monokaryotic fruiting initiates from large cells, which are induced by high temperature seed culture

**Figure 3:**
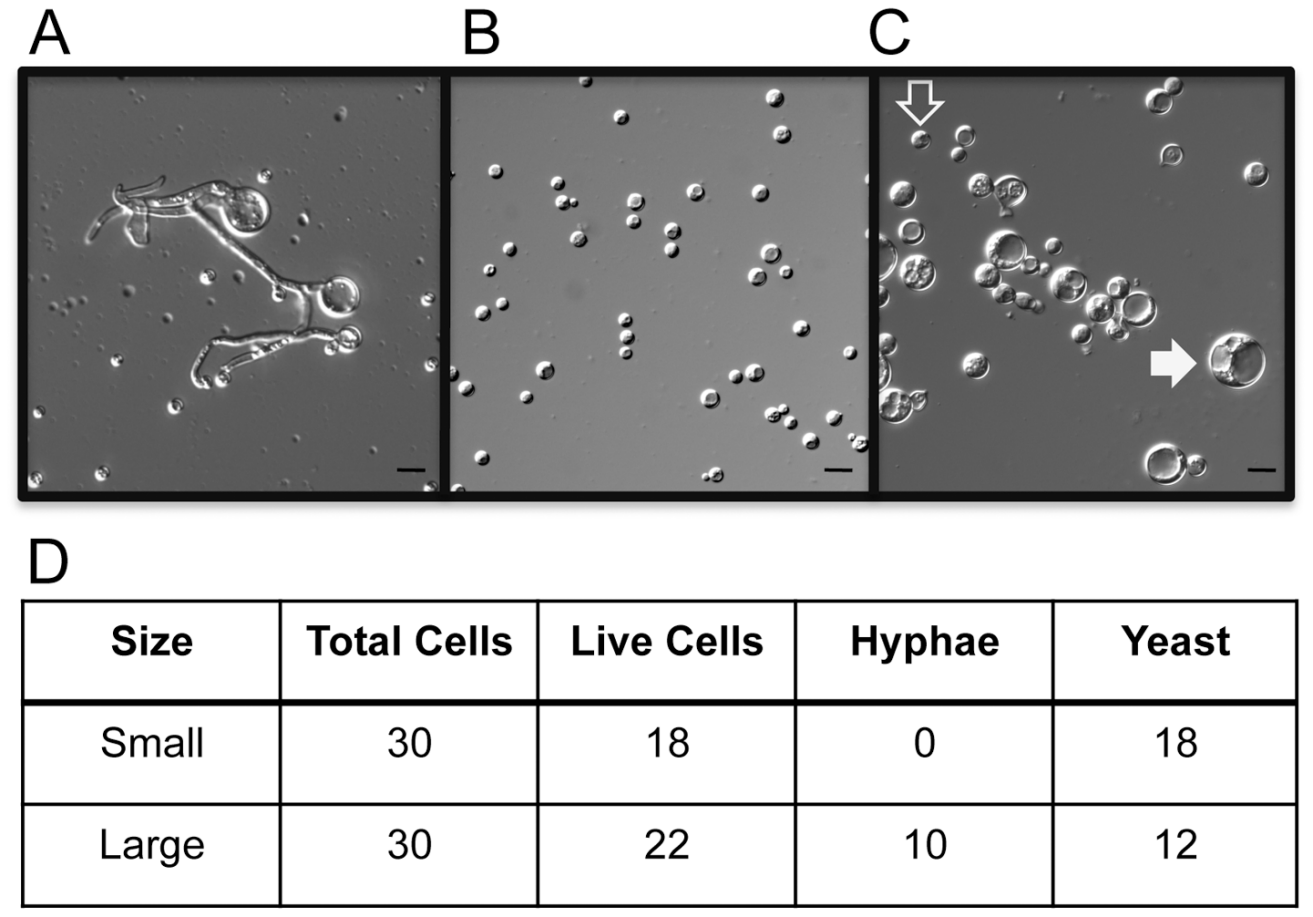
The effect of seed culture temperature on morphology. (A) Wet mount of cells scraped from filament agar after 24 h growth showing hyphae growing from an enlarged cell. (B) Wet mount of cells from YPD agar after 24 h growth at 30°C showing a homogenous population of normal-sized (2–5 µm) yeast cells. (C) Wet mount of cells scraped from YPD agar after 24 h growth at 37°C showing a mix of enlarged (filled white arrowhead) and normal-sized cells (open arrowhead). All images were obtained with DIC microscopy at 400× magnification. Bar = 10 µm. (D) Results of micromanipulation of enlarged and normal-sized cells from a 24 h, 37°C YPD agar culture scored for morphology after germinating on filament agar.

The production of enlarged cells in *C. neoformans* has been reported to occur when cells are exposed to opposite mating type cells in standard mating type mixes [11], and in confrontation assays, which are performed by streaking cells of opposite mating type in close proximity to each other [20]. Clinical studies have also found this cell type *in vivo* [16], [18], [21], [22]. Because only certain cells produced hyphae during the quantitative monokaryotic fruiting assay, we decided to determine if there were developmental differences among cells after high temperature seed culture. Microscopic observation of wet mounts prepared from cells scraped off of filament agar after growing as a seed culture at 37°C showed that filaments always originated from enlarged cells (Fig. 3A). When cells from 30°C and 37°C seed cultures were screened for enlarged cells, we only observed enlarged cells from the 37°C seed culture, although this incubation temperature produced both enlarged and smaller, normal-sized cells (Fig. 3B and 3C). To confirm that the enlarged cells were responsible for the production of hyphae during monokaryotic fruiting, large and small cells from a 37°C seed culture were micromanipulated onto filament agar and then monitored for hyphae production. Analysis of hyphae production from each single cell revealed that small cells only grew into yeast cells while the large cells grew as both yeast and hyphae (Fig. 3D). These results demonstrated that only a subset of the population grown at high temperature, which can be distinguished by the larger cell size, becomes competent to produce hyphae.

### Enlarged cells are haploid, arrested in G2, and produce haploid hyphae

The need for enlarged cells prior to the initiation of monokaryotic fruiting suggested that the mechanism for production of this phenotype possibly involved changes in cell ploidy since yeast ploidy has been noted to be associated with cell size [23]. In *C. neoformans*, monokaryotic fruiting has been shown to result in ploidy changes of yeast cells produced specifically from the hyphal filaments [13]. These observations suggested to us that monokaryotic fruiting may occur through ploidy changes, which are manifested as enlarged cells that arise from an endoduplication event, as has been previously suggested [24].

**Figure 4:**
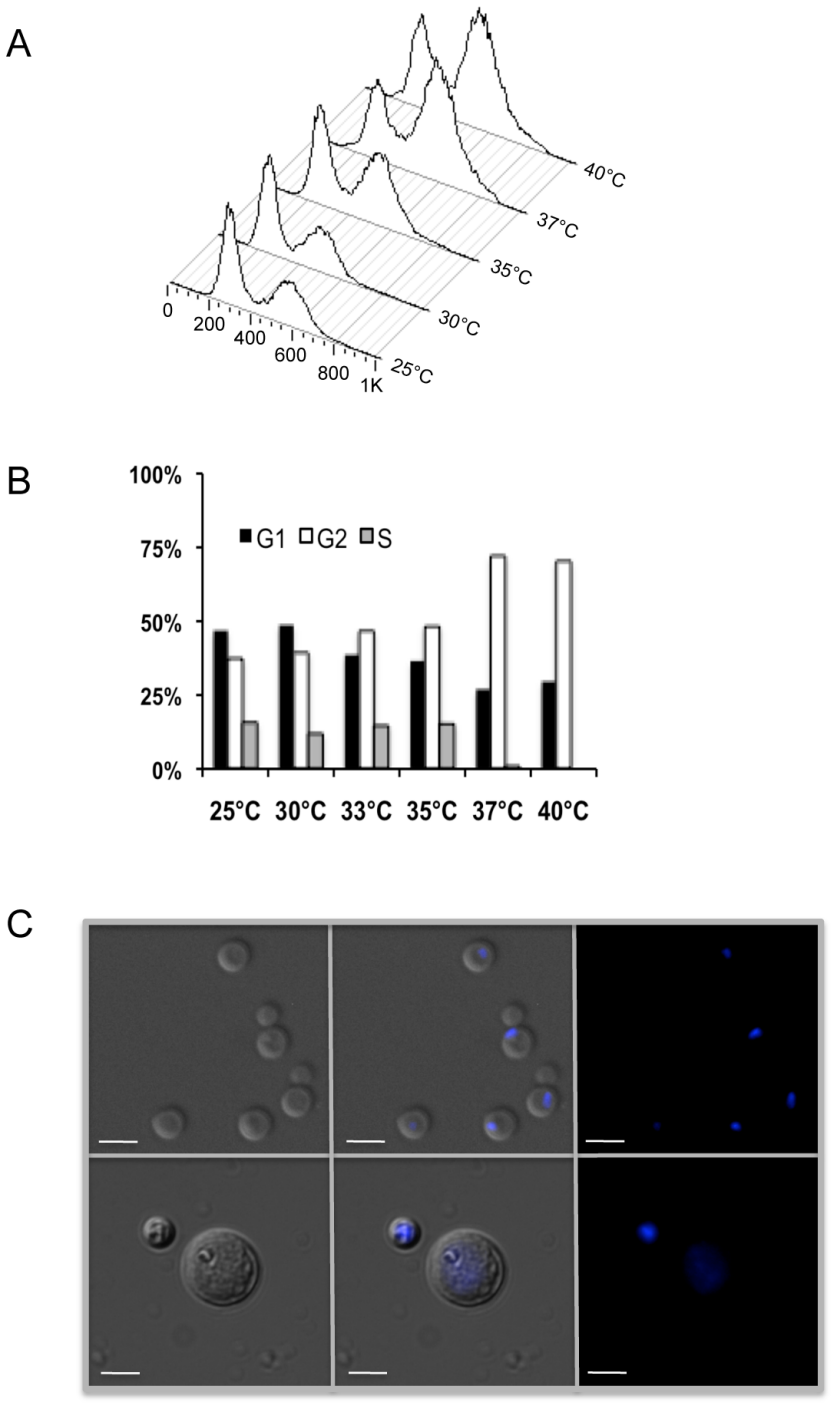
Monokaryotic fruiting originates from haploid cells. (A) Cell cycle analysis of cells grown at various temperatures reveals that only peaks corresponding to a DNA content of 1N and 2N were observed, ruling out the formation of diploids. Peaks represent cell number. Scale represents propidium iodine fluorescent intensity. (B) Cell cycle analysis of JEC-21 grown at different temperatures on YPD agar for 24 hrs shows that with increasing temperature, the percentage of cells arrested at G2 increases. (C) DAPI stained (0.1 µg/ml) nuclei of control and G2-arrested cells. The top panel shows control cells grown at 30°C using DIC/Normarsky, DIC/Normarsky with DAPI staining, and the merged image. These cells were normal-sized with visibly stained nuclei. In contrast, the bottom panel of three images shows cells grown at 37°C, which yielded large and small cells. The size of the smaller cells was consistent with the control cells grown at 30°C. The staining pattern of these cells was also consistent with the control cell staining pattern in which the nuclei stained more compactly than the nuclei of the larger cells, which stained larger, but more diffuse. Cells were visualized at 400×. Bar = 5 µm.

Cell cycle analysis of seed cultures grown at different temperatures (25°C, 30°C, 35°C, 37°C and 40°C) all revealed only 1n and 2n DNA content peaks, with no evidence of a 4n peak (Fig. 4A). This result showed that there was no ploidy change after high temperature (35°C, 37°C, 40°C) seed culture growth, demonstrating that the enlarged cells, which were responsible for monokaryotic fruiting, were haploid. Additionally, we found that as seed culture temperature increased from 25°C to 40°C, the percentage of cells in G1 decreased, the percentage of cells in G2 increased, and the percentage of cells in S phase was similar until dropping almost to 0 at 37°C (Fig. 4B). DAPI staining confirmed the relationship between cell size and G2 arrest as the staining patterns of large and small cells differed (Fig. 4C). The smaller cells displayed a compact nuclear staining pattern while the larger cells displayed a larger, diffuse staining pattern, which has been observed in other G2-arrested fungi [25], [26]. These results demonstrated that the effect of increasing temperature on monokaryotic fruiting involved the cell cycle, specifically G2 arrest.

**Figure 5:**
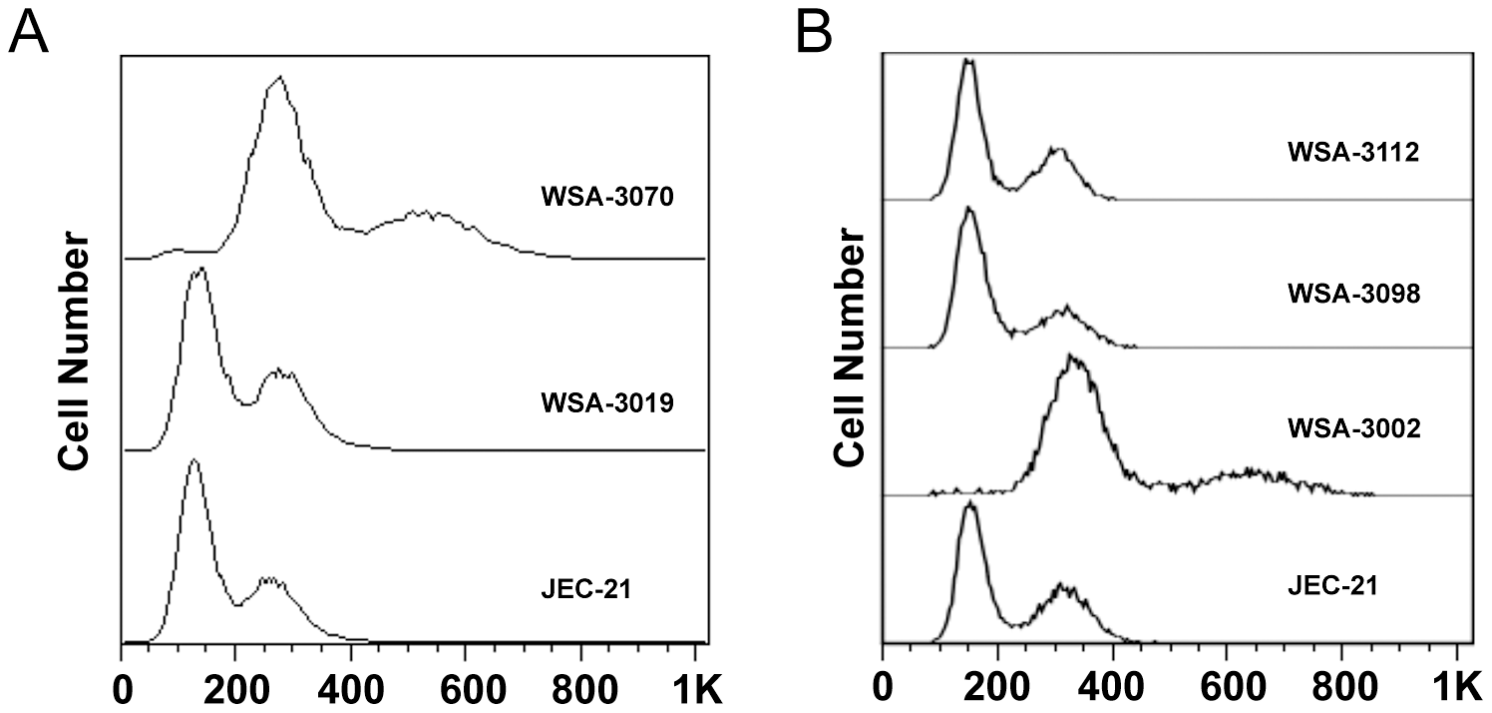
FACS analysis of hyphal blastospores. (A) Blastospores recovered from hyphae produced during monokaryotic fruiting. JEC-21 is the haploid control strain used for the fruiting assays. WSA-3070 is one of two blastospores out of seventy-eight total blastospores recovered from the blastospore assay, which was found to be diploid, as evidenced by comparison to the JEC-21 FACS profile. WSA-3019 is one of the seventy-six blastospores recovered from the same assay, which was found to be haploid, as evidenced by comparison to the JEC-21 FACS profile. (B) FACS profiles of blastospores recovered from hyphae with and without basidiospore chains. Representative profile of a blastospore (WSA-3112) recovered from a hypha without basidiospores and a profile of a blastospore (WSA-3098) from a hypha with basidiospores. Comparison of these two profiles to the control haploid (JEC-21) and diploid (WSA-3002) profiles revealed them both to be consistent with a haploid cell. These results suggest that if diploidization occurs in hyphae during monokaryotic fruiting, it occurs late or even after hyphae have formed, and may be restricted to the basidium.

In spite of the FACS results showing that hyphal-competent cells were haploid, we could not rule out a change in ploidy just before, or during growth in the hyphal phase. Unfortunately ploidy determination by FACS analysis of hyphae is physically restricted by the filamentous characteristics of the cells. However, hyphal ploidy can be determined using a blastospore assay [13]. The results of this assay revealed that 76 out of 78 blastospores (97%) were haploid, demonstrating that the hyphae produced during monokaryotic fruiting were haploid, which again excluded *α*-*α* mating or endoduplication as monokaryotic fruiting mechanisms in our system (Fig. 5A). As a further control, the two diploid blastospores were sub cultured repeatedly for an additional two weeks and then retested by FACS, which revealed that they remained diploid, ruling out the possibility that haploid blastospores could be segregation products of diploid blastospores. While a late endoduplication event could explain the two diploid spores, this possibility is unlikely since the 78 spores were picked from 78 independent hyphae. However based on the recovery of two diploid blastospores in our assay, and the rarity of basidiospore production during monokaryotic fruiting, we hypothesized that there could be two hyphal types produced during monokaryotic fruiting, which could be distinguished by ploidy. One type could be vegetative in nature and not undergo basidiosporogenesis (haploid) and a second type could be generated that ultimately produced basidiospores (diploid). To address these two possibilities, 37°C seed culture cells were spread onto filament agar and the resultant colonies screened for basidiospore chains. The blastospore assay was performed on blastospores recovered from hyphae with and without basidiospores. Cell cycle analysis again showed that all of the blastospores were haploid, regardless of whether or not the hypha produced basidiospores (Fig. 5B), which was consistent with our previous observations that excluded *α*-*α* mating or endoduplication as the mechanisms of monokaryotic fruiting. Together, these results demonstrate that endoduplication is not required for monokaryotic fruiting as these hyphae are produced from haploid cells and remain haploid, in spite of being able to occasionally generate diploid yeast cells.

### Monokaryotic fruiting proceeds only from G2-arrested cells

**Figure 6:**
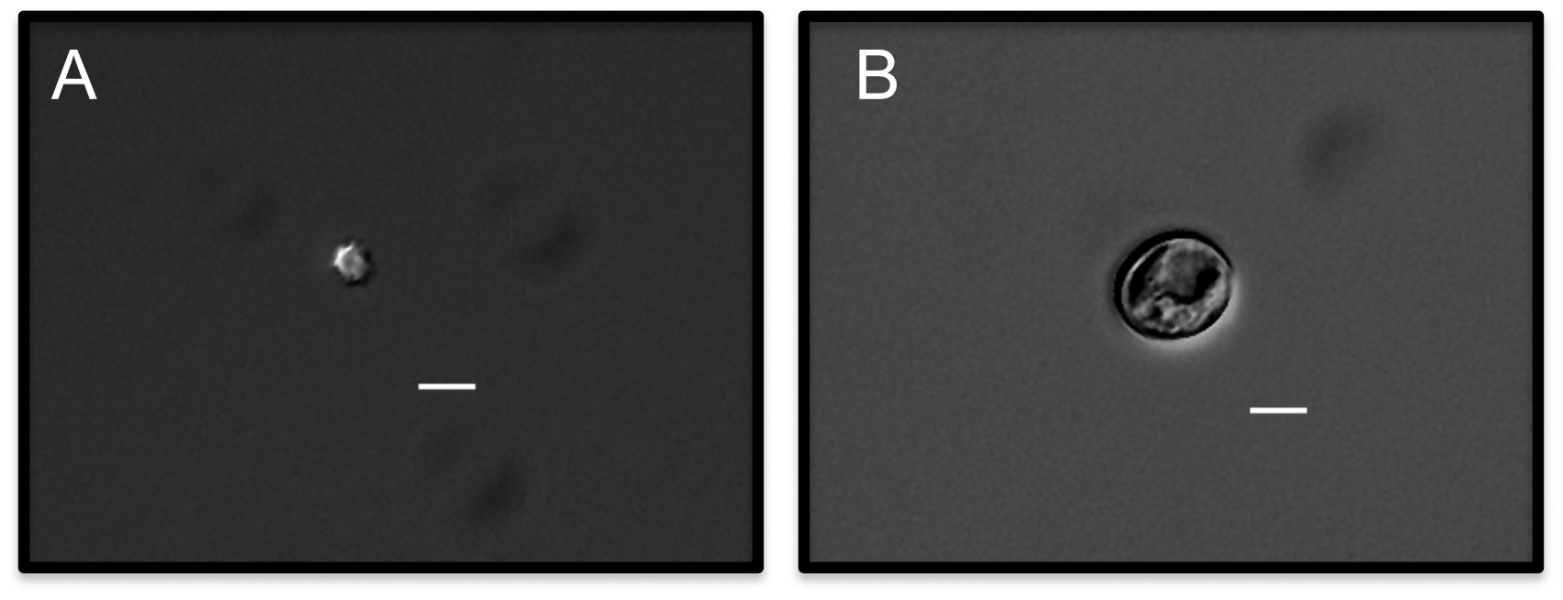
Size comparison of sorted G1 and G2 phase cells. JEC-21 cells were grown on YPD agar at 37°C for 24 hrs, then were fixed and stained with propidium iodide. Cells were then sorted according to DNA content and then visualized by DIC microscopy at 400× in order to compare their sizes. A). Sorted G1 cell fraction. B). Sorted G2 cell fraction. Microscopy showed that the G2 fraction contained cells approximately 4× the size of cells in the G1 fraction. Bar = 5 µm.

**Figure 7:**
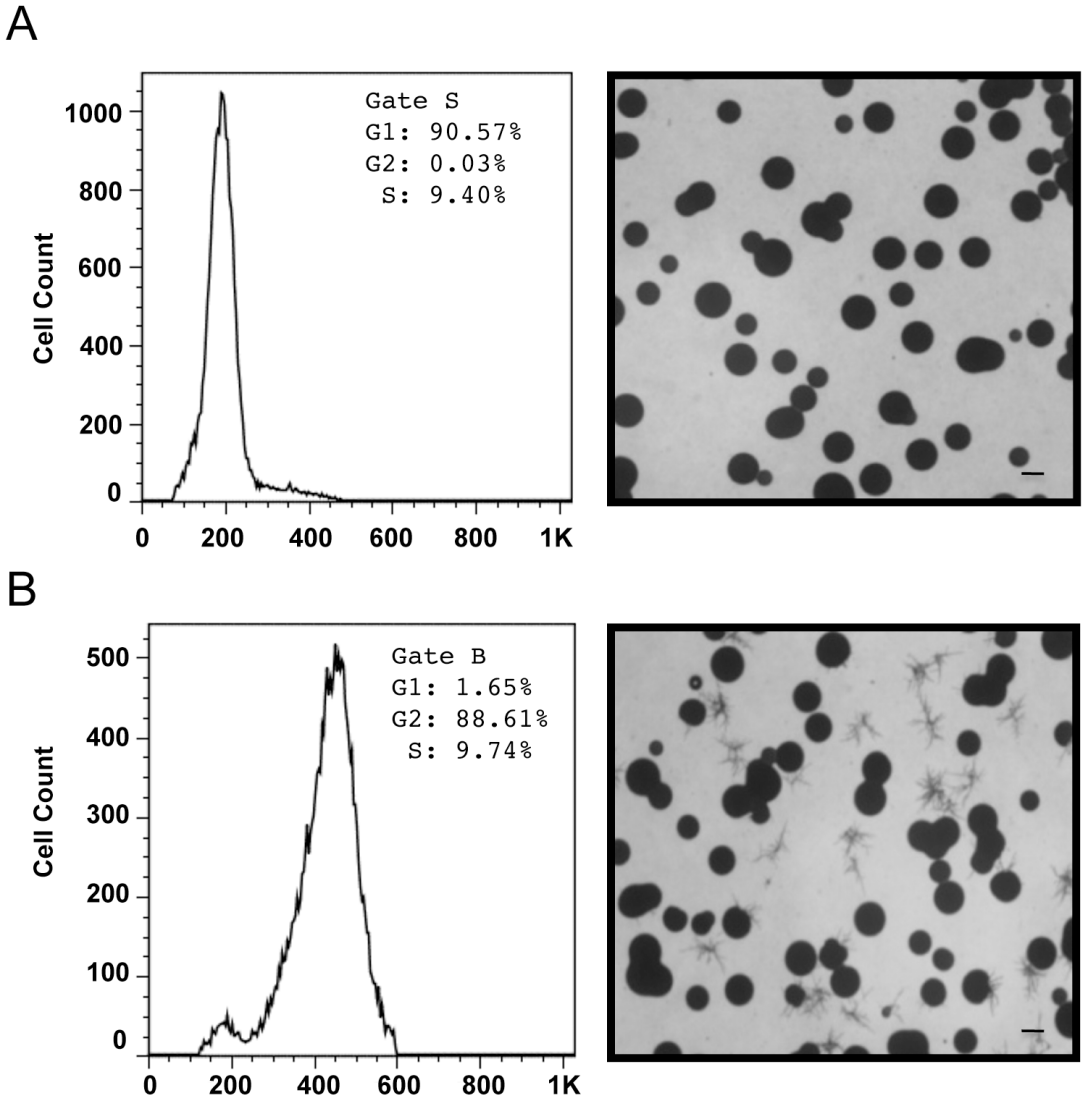
The relationship between cell size, cell cycle, and monokaryotic fruiting ability. (A) Cell cycle analysis of sorted JEC-21 cells revealed that the smaller cells were almost exclusively (90.57%) G1 phase cells and yeast-like in morphology when plated onto filament agar. (B) In contrast to the results observed for smaller cells, the population of enlarged cells recovered by sorting revealed that enlarged cells were primarily G2 phase (88.61%), and contained a sub population of cells (∼40–50%) able to undergo monokaryotic fruiting when these cells were plated onto filament agar. Approximately 20,000 cells were sorted for each cell size. Bar = 1.0 mm.

To determine whether only G2-arrested cells undergo monokaryotic fruiting, we sorted G1 and G2 phase, 37°C seed culture cells according to DNA content. Microscopic observation of sorted G1 and G2 cells revealed that G2 phase cells were much larger than G1 phase cells (Fig. 6). We next sorted live 37°C seed culture cells according to cell size and performed cell cycle analysis on the two populations. The results indicated that the smaller cells had a single DNA content peak at the 1n position while the enlarged cells had a single DNA content peak at the 2n position, which lead us to conclude that the small cells were G1 phase cells and the large cells were cells in G2 arrest (Fig. 7). The two populations were then assayed for monokaryotic fruiting ability, which revealed that monokaryotic fruiting was a property only of the G2 fraction (Fig. 7).

**Figure 8:**
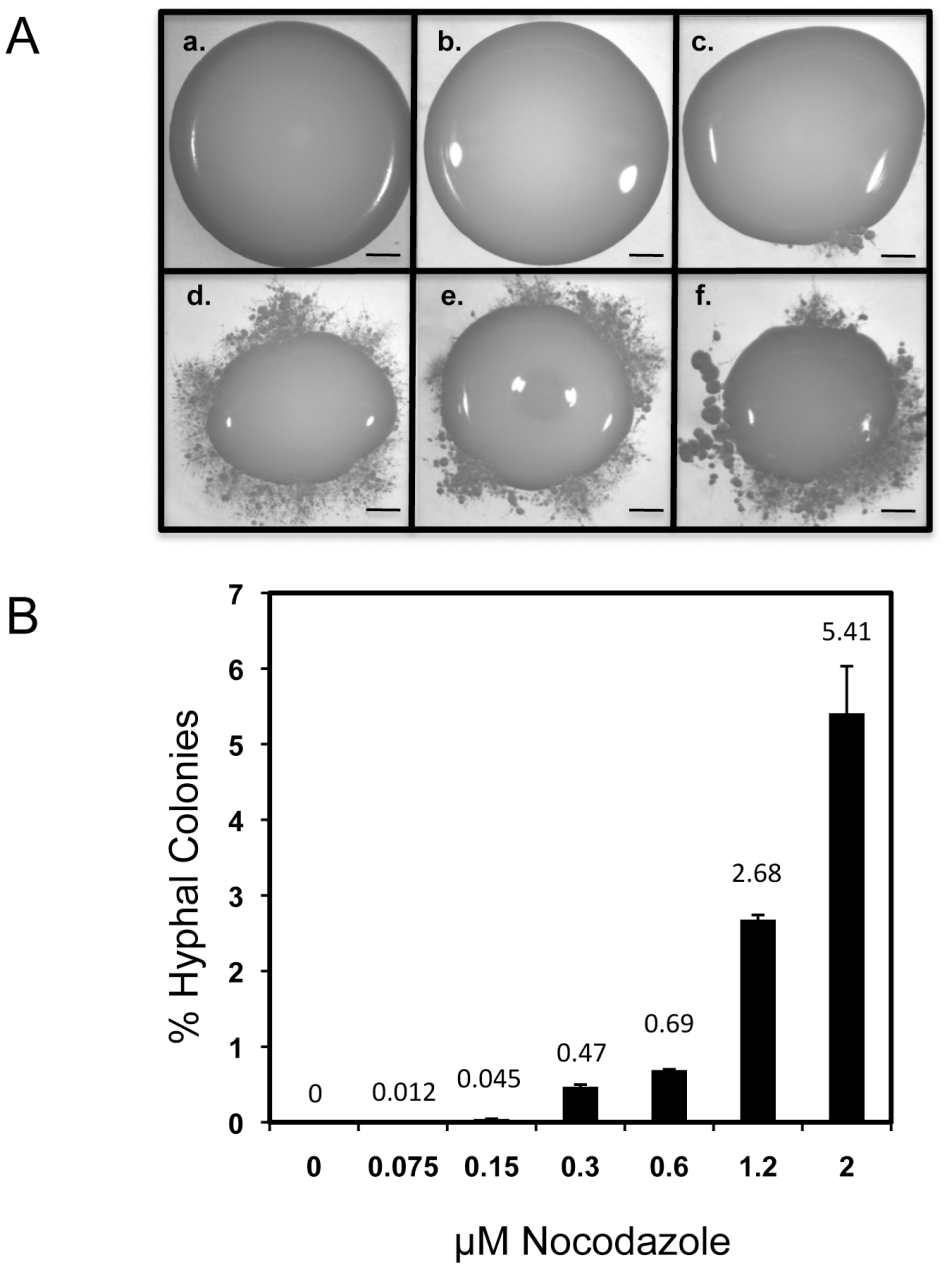
The effect of nocodazole treatment on monokaryotic fruiting. JEC-21 cells were cultured in YPD broth plus different concentrations of nocodazole at 30°C for 24 hrs, then dropped onto filament agar and incubated at 25°C for 5 days. (A) Qualitative agar assay for monokaryotic fruiting. Nocodazole concentrations consisted of a.) 0 µM, b.) 0.075 µM, c.) 0.15 µM, d.) 0.30 µM, e.) 0.60 µM, f.) 1.20 µM. Hyphal production begins at nocodazole concentrations ≥0.15 µM. Bar = 0.5 mm. (B) Quantitative agar assay for monokaryotic fruiting. Concentrations of nocodazole are indicated. The percent of colonies undergoing monokaryotic fruiting was calculated from the total colonies that grew out on the agar plates. Plates were assayed in triplicate.

As a final confirmation that monokaryotic fruiting requires G2 arrest, cells were treated with nocodazole, a G2/M arrest agent that inhibits and disassembles microtubules [27]. Cells were grown as seed cultures at 30°C (the non-permissive seed culture temperature), then assayed for monokaryotic fruiting ability. The experiment showed that cells treated with nocodazole became competent to produce hyphae on filament agar in a dose-dependent manner even though they were grown as a seed culture under conditions that did not normally lead to monokaryotic fruiting, whereas untreated cells only grew as yeasts (Fig. 8). Taken together, these results demonstrate that G2-arrested cells can serve as a starting point for cells that undergo monokaryotic fruiting.

## Discussion

In this study we have identified a novel mechanism in *C. neoformans* that leads to the production of hyphae, with or without basidiospores, by haploid cells (monokaryotic fruiting). This mechanism appears to be dependent on the cell cycle and initiates from cells in G2 arrest. It occurs in the absence of *α*-*α* mating and/or endoduplication, thereby demonstrating that monokaryotic fruiting can occur asexually. Previous studies have shown that sexual reproduction can occur between cells of the same mating type, resulting in monokaryotic fruiting [13], and that this phenomenon occurs in nature [24]. Under the specific conditions of this study, notably a 37°C seed culture temperature, we saw no evidence of an *α*-*α* cell fusion event, nor did we find evidence of endoduplication within the hyphae even though we screened hyphae that had produced basidiospores. During *C. neoformans* basidiosporogenesis, sexual reproduction results in meiosis in the basidium followed by successive mitotic divisions that yield the nuclei, which ultimately are inserted into spores as they form on the basidial surface [20]. Lin *et al.* observed that when fruiting was derived from an *α*-*α* fusant, sporogenesis was robust with spore chains that were long and phenotypically similar to *α-a* mating during sexual reproduction [13]. This process was found to be impaired in *dmc1* mutants, which are meiotic mutants that still produce spores, but at a much lower frequency than sexually produced spores, and with truncated spore chains that sometimes occur as dyads (two rather than four chains) [13]. The phenotype of basidiospores produced in the *dmc1* strains was strikingly similar to what we observed in this study and what was previously reported [6]. These observations may suggest that basidiosporogenesis can occur mitotically without meiosis, although we cannot exclude a duplication event in the basidium immediately followed by meiosis and sporogenesis. We did, however, test a *dmc1* mutant and found that it was able to undergo monokaryotic fruiting under our conditions (data not shown).

Because our study was done in the same strain background as the study by Lin et al., we reviewed the conditions of both experiments and found some differences that may explain the contrasting differences in ploidy. Our study used a high temperature seed culture condition, which results in rapid hyphae production upon filament agar plating. The seed culture conditions in Lin's study were not clear, however, their plating medium was V8 agar, which is normally used for mating *C. neoformans*, and their incubation period was for a period of weeks, whereas we screened at 24 hrs and observed hyphae in as little as four hours, although cells also produced hyphae on V8 agar under our conditions. Both filament agar and V8 agar have high agar contents; however, V8 agar is an undefined medium with V8 juice as the basal ingredient. Filament agar, on the other hand, is a low-glucose, defined medium with Yeast Nitrogen Base without amino acids and without ammonium sulfate as the source of vitamins and cofactors. Although both media are starvation media, they are substantially different in composition, which may be one explanation for the differences in hyphal types that we observed. The seed culture conditions, or more precisely, the cell cycle stage may be another explanation.

Our initial investigation of the relationship between the cell cycle and monokaryotic fruiting focused on detecting what we presumed would be a transition to diploidy in yeast cells at some point during seed culture growth, which we believed would coincide with the appearance of enlarged cells in the seed culture and the association of this cell type with the hyphal progenitor. The high frequency of fruiting and enlarged cells in the seed culture suggested that detection of the diploidization event would be unambiguous. However, the data showed that instead of an *α*-*α* fusion or endoduplication event, which would result in a diploid cell, the actual mechanism that resulted in hyphae production was G2 arrest. The reason for the requirement of G2 arrest to induce hyphae is not clear, and while G2 arrest is required for monokaryotic fruiting, not all arrested cells produced hyphae. We suspect that the subpopulation of non-hyphal, G2-arrested cells consisted of cells that escaped G2, and then proceeded to bud rather than differentiate into a hypha. These two outcomes resemble the decision point that a pheromone-exposed, G2-arrested *Ustilago maydis* cell faces with regard to which of the two developmental paths it will follow (conjugation tube formation or budding) [26]. How the generation of occasional diploid blastospores occurs is also not clear. Given that *C. neoformans* hyphae produce typical basidiomycete-like clamp connections, the incomplete fusion of these structures in monokaryotic hyphae combined with an aberrant segregation event during the budding of blastospores off of the hyphal compartment may yield the diploid cells that we observed at low frequency.

Previous studies of the *C. neoformans* cell cycle have identified a number of stressors that cause G2 arrest, including oxygen depletion [28], stationary growth phase [29], and temperature [30]. Under our experimental conditions, hyphal competency occurs prior to transfer to hyphal inducing conditions (starvation on filament agar) and not during growth on filament agar. Therefore, stationary growth phase is not a factor nor is oxygen depletion since cultures were grown on the agar surface, and only for 24 hrs. Consequently, growth temperature seems to be responsible for inducing competency. Under our conditions, the 37°C incubation temperature differs from the original incubation temperature (30°C) for monokaryotic fruiting [6], which suggests that temperature is an important variable. In fact, while we did not see the temperature effect on all strains of *C. neoformans*, we were able to induce hyphae in all four serotypes. Interestingly, the reports of enlarged cells *in vivo* [16], [18], [21], [22] reflect growth at elevated temperature in the mammalian body. Other pathways have also been shown to influence *C. neoformans* cell size *in vitro*, including cAMP, RAS, and PKA [16], [31], [32], suggesting the possibility of conserved stimuli that may regulate these pathways. Presently, the *STE12α* signal transduction pathway seems to be the major or sole regulator of monokaryotic fruiting as this gene is required for monokaryotic fruiting regardless of inducing conditions. Fruiting still occurred normally in *cpkΔ1* (pheromone response pathway), and *cacΔ1* (cAMP pathway) mutants, ruling out these pathways as regulators of temperature-induced monokaryotic fruiting. Other pathways, such as the calcineurin signal transduction pathway cannot be ruled out, but are more complicated to test since some mutants in this pathway do not grow at high temperature [33].

A key characteristic of enlarged cells *in vivo* appears to be polyploidy, which has been hypothesized to arise when the M phase of the cell cycle is skipped [16], [18]. This enlarged cell phenotype appears to be a potential virulence factor as they are poorly phagocytized, if at all [18]. Perhaps increasing cell size evolved as a physical defense mechanism against predatory grazers, which in turn, protects cells from being phagocytized *in vivo* via the same mechanisms. It appears that the enlarged cell phenotype can be produced by multiple mechanisms: cell cycle arrest, *a*-*α* or *α*-*α* cell fusion, and endoduplication, each of which may have a different purpose. The developmental options available after cell cycle arrest may have been selected for in *C. neoformans* to enhance survival in its specific environmental niche while inadvertently creating an important human fungal pathogen. What remains to be determined is how high temperature generation of the large cell and monokaryotic fruiting phenotypes was incorporated into the evolution of *C. neoformans*. With the exception of *C. neoformans*, virtually all members of this genus grow poorly or not at all at mammalian ambient temperature. In contrast, all of the major human fungal pathogens grow at 37°C and virtually all of them have a hyphal phenotype. Perhaps the association of many basidiomycetes with rotting wood or decaying vegetation in general led to high temperature exposure and subsequent genetic selection during self-heating, compost-like conditions caused by microbial metabolism of organic matter. Once nutrients were consumed, hyphal extension towards additional nutrients and/or sporulation in the absence of nutrients could have completed the evolution of *C. neoformans* into a pathogen via development of a mechanism of infectious particle (basidiospores) dispersion combined with the ability to grow at elevated temperatures. Selection for the molecular linkage of pathways controlling cell cycle, nutrient sensing, and ultimately, differentiation, could have been the outcome of this lifestyle and allowed the fungus to coordinately regulate these pathways, thus enabling it to effectively exist as a saprophyte or pathogen.

## Materials and Methods

### Media and strains

YPD agar, MIN agar, V8 agar, and filament agar were prepared as described previously [6], [7], [11] with or without amino acids or nucleic acid supplements as required. JEC-21 is a wild type, *MATα* isolate that was used in the initial characterization of monokaryotic fruiting in *C. neoformans* [34]. WSA-79 is a serotype D clinical isolate from Maryland, WSA-522 is a serotype A clinical isolate from Thailand, WSA-533 is a serotype B environmental isolate from Australia, and WSA-2507 is a serotype C clinical isolate from Maryland. Additional strains, all of which were derived from the original JEC-21 - JEC-20 congenic pair [34], consisted of the following genotypes: WSA-1 (*MATα lys2*), WSA-70 (*MATα ade2 lys2*), WSA-591 (*MATα ade2*), WSA-1226 (*MATα ura5*), WSA-2126 (*MATα ura5 cpk1Δ::ura5*), WSA-3002 (*MATα/MATα* fusant from WSA-1226×WSA-70×WSA-68), WSA-3019 (*MATα* haploid blastospore), WSA-3070 (*MATα/MATα* diploid blastospore recovered from JEC-21 hypha without basidiospore chains), WSA-3098 (*MATα* haploid blastospore recovered from JEC-21 hypha with basidiospore chains), WSA-3112 (*MATα* haploid blastospore recovered from hypha without basidiospore chains), WSA-3145 (*MATα ura5 cpk1Δ::ura5 CPK1::NEO^r^*). *MATa* strains included WSA-65 (*MATa ura5 lys1 ade2*) and WSA-68 (*MATa ura5 ade2 lys2*).

#### Monokaryotic fruiting assay

The standard assay for monokaryotic fruiting was performed as described [11] using YPD agar as the medium and a seed culture incubation temperature of 37°C for 24 h unless otherwise indicated. Isolates were harvested from plates and then streaked or spread (after suspension in distilled water) onto the appropriate agar plate, which was then incubated at 25°C until cells were photographed using an Olympus BX51, bright field, Spot Idea Camera, (Diagnostic Instruments, Inc., Sterling Heights, MI).

#### Quantitative monokaryotic fruiting assay

Monokaryotic fruiting efficiency was determined by quantitative assay. This assay was performed by suspending ∼10^6^ cells from a YPD seed culture grown at 37°C in 1 ml sterile water. This suspension was then serially diluted to yield approximately 2000 cells per ml. A 100 µl aliquot was then spread onto filament agar, which was incubated at 25°C for 24 h. The number of hyphal colonies was then compared to the total number of cells to determine the percentage of cells that were capable of undergoing monokaryotic fruiting.

#### Hyphal ploidy assays

Hyphal ploidy was determined using the blastospore assay [13]. Single blastospores were recovered from hyphae produced by filament agar colonies using an Axiolab micromanipulator (Zeiss, Thornwood, New York). Each blastospore was isolated from independent hyphae-producing colonies, one blastospore per hypha. A second screen was used to compare blastospore ploidy from hyphae with and without basidiospores, which were part of the same colony. Colonies were identified that produced hyphae with basidiospores on a filament agar spread plate. Blastospores were then isolated from single hyphae that had produced four chains of basidiospores. Additional blastospores were also isolated from the same colony, but from single hyphae that had not produced basidiospores. All blastospores were immediately transferred to YPD agar and grown out for 72 hrs at 30°C. Each isolate was then screened for ploidy by FACS analysis as described below.

### Nocodazole treatment

Nocodazole (Sigma-Aldrich, St. Louis, MO) stock was prepared in DMSO at 1.5 mM and then used to prepare different dilutions in YPD broth (YPD-0.075 µm nocodazole, YPD-0.15 µm nocodazole, YPD-0.30 µm nocodazole, YPD-0.60 µm nocodazole, YPD-1.2 µm nocodazole). JEC-21 cells were added to 1.5 ml nocodazole-YPD broth in 15 ml snap cap tubes (BD Biosciences, Franklin Lakes, NJ), at a final concentration of 1×10^6^ cells/ml. Tubes were shaken at 200× RPM at 30°C for 24 hrs. Five µl of overnight culture were then dropped onto filament agar and incubated at 25°C for 5 days. The quantitative assay was performed by growing cells as above, and then plating cells onto filament agar as described in the quantitative monokaryotic fruiting assay. Pictures were taken with an Olympus SZX12 stereo microscope (Olympus, Center Valley, PA) at 2× magnification.

### Cell fusions

To perform *α*-*α* cell fusions, 1×10^6^ cells of complementary, auxotrophic, *MATα* strains were mixed, cultured on YPD agar at 37°C for 24 hrs, and then transferred onto filament agar plates. The plates were incubated at 25°C for 24 hrs. The mixture was scraped from the plate, suspended in 1.0 ml sterile H_2_O, and then 200 µl of cells from this suspension were spread onto MIN agar plates, which were then incubated at 30°C for 4 days to screen for fusants. Assisted mating reactions, in which two *MATα* strains were induced to fuse by including a *MATa* helper strain, were performed according to previously described methods [19]. Plates were photographed at 0.5× magnification.

### Flow cytometry and cell sorting

Cells were harvested, fixed, and stained with propidium iodide (Sigma-Aldrich, St. Louis, MO), and then sorted as described by Sia et al. [35]. Cell cycle analysis was performed using a BD FACSCalibur flow cytometer (Becton Dickinson Biosciences, Sparks, MD). CellQuest Pro software was used for cell collection, and data analysis was performed using ModFit and FlowJo. G1, S, and G2 phases were identified using the Dean-Jett-Fox mathematical model. G2-arrested cells were identified as described [25], [26], [36], [37], [38]. This population of cells typically shows a large cell phenotype, an increased proportion of cells with 2C DNA content when compared to controls, and a DAPI staining pattern that shows larger nuclei vs. smaller condensed nuclei of G2 phase cells (see below). Cell sortings were performed on a BD FACSAria III (Beckton Dickinson Biosciences) cell sorter and analyzed using BD FACSDiva 6.1 software. Aliquots of sorted live cells were also used to perform the quantitative monokaryotic fruiting assay. All FACS analysis was performed at the Flow Cytometry Core Laboratory at The University of Texas Health Science Center at San Antonio.

### Microscopy

Cells were stained with DAPI according to the method described by Fuchs *et al.* [39]. Briefly, 1×10^7^ cells were harvested from 24 h YPD agar plates, which were grown at either 30°C or 37°C, washed twice with phosphate buffer (0.1 M KH_2_PO_4_, 1.25 mM EGTA, 1.25 mM MgCl_2_ at pH 6.9), then resuspended in 400 µl fixative (5% paraformaldehyde in wash buffer) followed by incubation at room temperature for 90 minutes. The cells were then washed three times in wash buffer and incubated with Lysing Enzymes (*Trichoderma harzianum*, Sigma-Aldrich, 1 mg/ml in sterile distilled water) for 20 min at 37°C. The cells were then washed once with sterile water, gently resuspended in 400 µl 0.3% Triton X-100 (Sigma-Aldrich), and permeabilized by incubation at room temperature for 15 minutes. The suspension was then pelleted and washed three times with PBS. Cells were stained in DAPI (Sigma-Aldrich) (0.1, 0.2, or 0.5 µg/ml) for 15 minutes, washed twice with PBS, and then resuspended in 200 µl prior to visualization.

Images were captured on a Zeiss AxioImager Z1 microscope (Carl Zeiss Microscopy, LLC, Thornwood, NY) equipped with an AxioCam MR3_2 CCD camera, using the filters for DAPI (365 nm excitation, 395 nm beam splitter, 420–470 nm emission filter) and differential interference contrast (DIC, Nomarski contrast). Image analysis and adjustments were performed using Axiovision software (Zeiss, Version 4.8). Images were adjusted only for frame alignment (to overlay DAPI over DIC), brightness, and contrast (adjustment of +0.01 contrast units over default), and all images received the same treatment.

## References

[1] Perfect JR (2006) *Cryptococcus neoformans:* a Sugar Coated Killer. In: Heitman J, Filler SG, Edwards JE, Mitchell AP, editors. Molecular Principles of Fungal Pathogenesis. Washington, DC: American Society for Microbiology. pp. 281–304.

[2] Waters L, Nelson M (2005) Cryptococcal disease and HIV infection. Expert Opinion on Pharmacotherapy 6: 2633–2644. 16316302 10.1517/14656566.6.15.2633

[3] Byrnes EJ 3rd, Marr KA (2011) The outbreak of *Cryptococcus gattii* in Western North America: Epidemiology and clinical issues. Current Infectious Disease Reports 13: 256–261. 21461678 10.1007/s11908-011-0181-0 PMC4696060

[4] Kwon-Chung KJ, Bennett JE (1978) Distribution of *a* and *alpha* mating types of *Cryptococcus neoformans* among natural and clinical isolates. American Journal of Epidemiology 108: 337–340. 364979 10.1093/oxfordjournals.aje.a112628

[5] Kwon-Chung KJ, Varma A (2006) Do major species concepts support one, two or more species within *Cryptococcus neoformans* ? FEMS Yeast Res 6: 574–587. 16696653 10.1111/j.1567-1364.2006.00088.x

[6] Wickes BL, Mayorga ME, Edman U, Edman JC (1996) Dimorphism and haploid fruiting in *Cryptococcus neoformans*: association with the *alpha*-mating type. Proc Natl Acad Sci U S A 93: 7327–7331. 8692992 10.1073/pnas.93.14.7327 PMC38983

[7] Clarke DL, Woodlee GL, McClelland CM, Seymour TS, Wickes BL (2001) The *Cryptococcus neoformans STE11alpha* gene is similar to other fungal mitogen-activated protein kinase kinase kinase (MAPKKK) genes but is mating type specific. Mol Microbiol 40: 200–213. 11298287 10.1046/j.1365-2958.2001.02375.x

[8] Wang P, Nichols CB, Lengeler KB, Cardenas ME, Cox GM, et al (2002) Mating-type-specific and nonspecific PAK kinases play shared and divergent roles in *Cryptococcus neoformans* . Eukaryot Cell 1: 257–272. 12455960 10.1128/EC.1.2.257-272.2002 PMC118036

[9] Davidson RC, Nichols CB, Cox GM, Perfect JR, Heitman J (2003) A MAP kinase cascade composed of cell type specific and non-specific elements controls mating and differentiation of the fungal pathogen *Cryptococcus neoformans* . Mol Microbiol 49: 469–485. 12828643 10.1046/j.1365-2958.2003.03563.x

[10] Wang P, Perfect JR, Heitman J (2000) The G-protein beta subunit *GPB1* is required for mating and haploid fruiting in *Cryptococcus neoformans* . Molecular and Cellular Biology 20: 352–362. 10594037 10.1128/mcb.20.1.352-362.2000 PMC85090

[11] Fu J, Mares C, Lizcano A, Liu Y, Wickes BL (2011) Insertional mutagenesis combined with an inducible filamentation phenotype reveals a conserved *STE50* homologue in *Cryptococcus neoformans* that is required for monokaryotic fruiting and sexual reproduction. Mol Microbiol 79: 990–1007. 21299652 10.1111/j.1365-2958.2010.07501.x

[12] Hull CM, Heitman J (2002) Genetics of *Cryptococcus neoformans* . Annual Review of Genetics 36: 557–615. 10.1146/annurev.genet.36.052402.152652 12429703

[13] Lin X, Hull CM, Heitman J (2005) Sexual reproduction between partners of the same mating type in *Cryptococcus neoformans* . Nature 434: 1017–1021. 15846346 10.1038/nature03448

[14] Lin X, Litvintseva AP, Nielsen K, Patel S, Floyd A, et al (2007) *alpha* AD *alpha* hybrids of *Cryptococcus neoforman*s: evidence of same-sex mating in nature and hybrid fitness. PLoS Genetics 3: 1975–1990. 17953489 10.1371/journal.pgen.0030186 PMC2042000

[15] Lin X, Huang JC, Mitchell TG, Heitman J (2006) Virulence attributes and hyphal growth of *Cryptococcus neoformans* are quantitative traits and the *MATalpha* allele enhances filamentation. PLoS Genetics 2: e187. 17112316 10.1371/journal.pgen.0020187 PMC1636697

[16] Zaragoza O, Garcia-Rodas R, Nosanchuk JD, Cuenca-Estrella M, Rodriguez-Tudela JL, et al (2010) Fungal cell gigantism during mammalian infection. PLoS Pathog 6: e1000945. 20585557 10.1371/journal.ppat.1000945 PMC2887474

[17] Garcia-Rodas R, Casadevall A, Rodriguez-Tudela JL, Cuenca-Estrella M, Zaragoza O (2011) *Cryptococcus neoformans* Capsular Enlargement and Cellular Gigantism during *Galleria mellonella* Infection. PLoS One 6: e24485. 21915338 10.1371/journal.pone.0024485 PMC3168503

[18] Okagaki LH, Strain AK, Nielsen JN, Charlier C, Baltes NJ, et al (2010) Cryptococcal cell morphology affects host cell interactions and pathogenicity. PLoS Pathog 6: e1000953. 20585559 10.1371/journal.ppat.1000953 PMC2887476

[19] Hull CM, Davidson RC, Heitman J (2002) Cell identity and sexual development in *Cryptococcus neoformans* are controlled by the mating-type-specific homeodomain protein *Sxi1alpha* . Genes & Development 16: 3046–3060. 12464634 10.1101/gad.1041402 PMC187491

[20] McClelland CM, Chang YC, Varma A, Kwon-Chung KJ (2004) Uniqueness of the mating system in *Cryptococcus neoformans* . Trends in Microbiology 12: 208–212. 15120139 10.1016/j.tim.2004.03.003

[21] Love GL, Boyd GD, Greer DL (1985) Large *Cryptococcus neoformans* isolated from brain abscess. Journal of Clinical Microbiology 22: 1068–1070. 3905847 10.1128/jcm.22.6.1068-1070.1985 PMC271885

[22] Cruickshank JG, Cavill R, Jelbert M (1973) *Cryptococcus neoformans* of unusual morphology. Applied Microbiology 25: 309–312. 4121033 10.1128/am.25.2.309-312.1973 PMC380794

[23] Galitski T, Saldanha AJ, Styles CA, Lander ES, Fink GR (1999) Ploidy regulation of gene expression. Science 285: 251–254. 10398601 10.1126/science.285.5425.251

[24] Lin X, Patel S, Litvintseva AP, Floyd A, Mitchell TG, et al (2009) Diploids in the *Cryptococcus neoformans* serotype A population homozygous for the alpha mating type originate via unisexual mating. PLoS Pathog 5: e1000283. 19180236 10.1371/journal.ppat.1000283 PMC2629120

[25] Al-Feel W, DeMar JC, Wakil SJ (2003) A *Saccharomyces cerevisiae* mutant strain defective in acetyl-CoA carboxylase arrests at the G2/M phase of the cell cycle. Proc Natl Acad Sci U S A 100: 3095–3100. 12626751 10.1073/pnas.0538069100 PMC152252

[26] Garcia-Muse T, Steinberg G, Perez-Martin J (2003) Pheromone-induced G2 arrest in the phytopathogenic fungus *Ustilago maydis* . Eukaryot Cell 2: 494–500. 12796294 10.1128/EC.2.3.494-500.2003 PMC161457

[27] Jacobs CW, Adams AE, Szaniszlo PJ, Pringle JR (1988) Functions of microtubules in the *Saccharomyces cerevisiae* cell cycle. The Journal of Cell Biology 107: 1409–1426. 3049620 10.1083/jcb.107.4.1409 PMC2115239

[28] Ohkusu M, Raclavsky V, Takeo K (2001) Deficit in oxygen causes G(2) budding and unbudded G(2) arrest in *Cryptococcus neoformans* . FEMS Microbiology Letters 204: 29–32. 11682173 10.1111/j.1574-6968.2001.tb10857.x

[29] Takeo K, Tanaka R, Miyaji M, Nishimura K (1995) Unbudded G2 as well as G1 arrest in the stationary phase of the basidiomycetous yeast *Cryptococcus neoformans* . FEMS Microbiology Letters 129: 231–235. 7607405 10.1111/j.1574-6968.1995.tb07585.x

[30] Takeo K, Ohkusu M, Kawamoto S (2003) Effects of growth temperature upshift on cell cycle progression in *Cryptococcus neoformans* . Mycoscience 44: 465–471.

[31] D'Souza CA, Alspaugh JA, Yue C, Harashima T, Cox GM, et al (2001) Cyclic AMP-dependent protein kinase controls virulence of the fungal pathogen *Cryptococcus neoformans* . Molecular and Cellular Biology 21: 3179–3191. 11287622 10.1128/MCB.21.9.3179-3191.2001 PMC86952

[32] Waugh MS, Nichols CB, DeCesare CM, Cox GM, Heitman J, et al (2002) Ras1 and Ras2 contribute shared and unique roles in physiology and virulence of *Cryptococcus neoformans* . Microbiology 148: 191–201. 11782511 10.1099/00221287-148-1-191

[33] Kraus PR, Nichols CB, Heitman J (2005) Calcium- and calcineurin-independent roles for calmodulin in *Cryptococcus neoformans* morphogenesis and high-temperature growth. Eukaryot Cell 4: 1079–1087. 15947200 10.1128/EC.4.6.1079-1087.2005 PMC1151996

[34] Kwon-Chung KJ, Edman JC, Wickes BL (1992) Genetic association of mating types and virulence in *Cryptococcus neoformans* . Infection and Immunity 60: 602–605. 1730495 10.1128/iai.60.2.602-605.1992 PMC257671

[35] Sia RA, Lengeler KB, Heitman J (2000) Diploid strains of the pathogenic basidiomycete *Cryptococcus neoformans* are thermally dimorphic. Fungal Genetics and Biology 29: 153–163. 10882532 10.1006/fgbi.2000.1192

[36] Garcia-Muse T, Steinberg G, Perez-Martin J (2004) Characterization of B-type cyclins in the smut fungus *Ustilago maydis*: roles in morphogenesis and pathogenicity. J Cell Sci 117: 487–506. 14679309 10.1242/jcs.00877

[37] Heimel K, Scherer M, Vranes M, Wahl R, Pothiratana C, et al (2010) The transcription factor Rbf1 is the master regulator for b-mating type controlled pathogenic development in *Ustilago maydis* . PLoS Pathog 6: e1001035. 20700446 10.1371/journal.ppat.1001035 PMC2916880

[38] Sgarlata C, Perez-Martin J (2005) The cdc25 phosphatase is essential for the G2/M phase transition in the basidiomycete yeast *Ustilago maydis* . Mol Microbiol 58: 1482–1496. 16313631 10.1111/j.1365-2958.2005.04925.x

[39] Fuchs BB, Tang RJ, Mylonakis E (2007) The temperature-sensitive role of *Cryptococcus neoformans ROM2* in cell morphogenesis. PLoS One 2: e368. 17426816 10.1371/journal.pone.0000368 PMC1838519

